# Medication adherence and self-care behaviours among patients with type 2 diabetes mellitus in Ghana

**DOI:** 10.1371/journal.pone.0237710

**Published:** 2020-08-21

**Authors:** Richard Adongo Afaya, Victoria Bam, Thomas Bavo Azongo, Agani Afaya, Abigail Kusi-Amponsah, James Mbangbe Ajusiyine, Tahiru Abdul Hamid

**Affiliations:** 1 Department of Nursing, Faculty of Allied Health Sciences, Kwame Nkrumah University of Science and Technology, Kumasi, Ghana; 2 Department of Public Health, School of Allied Health Sciences, University for Development Studies, Tamale, Ghana; 3 Department of Nursing, School of Nursing and Midwifery, University of Health and Allied Sciences, Ho, Ghana; 4 Department of Maternal and Child Health, School of Nursing, University of Ghana, Accra, Ghana; 5 Department of Trauma and Orthopaedics, Tamale Teaching Hospital, Tamale, Ghana; Brown University, UNITED STATES

## Abstract

**Background:**

Diabetes often coexists with other medical conditions and is a contributing cause of death in 88% of people who have it. The study aimed at evaluating medication adherence, self-care behaviours and diabetes knowledge among patients with type 2 diabetes mellitus in Ghana.

**Methods:**

A total of 330 participants were recruited into the study from three public hospitals in the Tamale metropolis. A validated medication adherence questionnaire and the Summary of Diabetes Self-care Activities tool were used to assess medication adherence and self-care activities respectively. Logistic and linear regressions were used to determine factors positively associated with non-adherence to medication and self-care behaviours respectively.

**Results:**

Of the 330 participants whose data were analysed, the mean (SD) age was 57.5 (11.8) years. The majority (84.5%) were adherent to anti-diabetes medication. Participant’s age, educational level, and practice of self-care behaviours influenced adherence to anti-diabetes medication. Participants aged 70 years and above were 79% less likely to be non-adherent to medication as compared to those below 50 years [OR = 0.21 (95%CI: 0.06–0.74), p = 0.016]. Participants with senior high school education were 3.7 times more likely to be non-adherent to medication than those with tertiary education [OR = 3.68 (95%CI: 1.01–13.44), p = 0.049]. Participants with tertiary education had an increase in the level of practice of self-management by 1.14 (p = 0.041). A unit increase in knowledge score also increased the level of practice of self-management by 3.02 (p<0.001).

**Conclusion:**

The majority of participants were adherent to anti-diabetes medication. Non-adherence to medication was associated with younger age and low level of education. Interventions to improve adherence should target younger and newly diagnosed patients through aggressive counselling to address healthy self-management behaviours.

## Background

Diabetes mellitus (DM) is an emerging lifestyle disease and is now an important global public problem and a silent killer [1]. Diabetes often coexists with other medical conditions and is a contributing cause of death in 88% of people who have it [2, 3]. The ravages of diabetes mellitus are estimated to affect about 625 million people by 2045 [4]. Globally, type 2 diabetes mellitus (T2DM) is the most common form of DM accounting for more than 90% of cases [1]. Its escalating incidence in combination with high rates of morbidity and mortality imposes a significant burden on the healthcare system and reduces the quality of life of those affected [5]. A recent systematic review and meta-analysis indicated that Ghana has a prevalence rate of 6.4% in adults [6]. If measures are not taken to improve diabetes management, the prevalence of the disease will continue to rise.

The ultimate aim of diabetes management is to attain optimal glycaemic control and prevent complications [2]. This requires that the patients incorporate various therapeutic interventions including adherence to medications, diet, blood sugar testing, and physical activity [7]. Adherence to self-care activities is complex and challenging with 98% of care being solely the responsibility of the patient [7]. Many studies have reported poor adherence to self-care behaviours [1, 8] and low medication adherence [2, 9]. Poor adherence to self-care behaviours and medications could lead to uncontrolled diabetes and subsequently the development of diabetes complications [1]. Numerous earlier studies that assessed medication adherence reported varied results. Studies from Uganda [10], Ethiopia [11], and United Arab Emirates [12] have reported medication adherence rates to be 83.3%, 85.1%, and 84% respectively. Inversely, studies in Switzerland and Botswana established low adherence rates of 40% and 52% respectively [13, 14]. Factors including older age, longer duration of diabetes, lack of finances, higher education, among others have been found to be significantly associated with medication adherence [1, 7, 9].

Diabetes knowledge is crucial in enhancing patients’ adherence to treatment [1]. Evidence from previous studies has indicated that having adequate knowledge about DM influences good self-care practices and attenuate diabetes complications tremendously [1, 15]. The patients’ knowledge about diabetes does not only promote self-management but provides them the ability to effectively adhere to treatment. It is very necessary to continue to assess adherence level to medication and self-care behaviours in patients with type 2 diabetes mellitus. This will enhance health care providers’ identification of patients with poor medication adherence and assist in planning appropriate strategies to promote medication and self-care adherence.

It seems evident from the review of relevant literature that similar studies have been conducted in Europe [13], Middle East [8, 12], South Africa [14], East Africa [11, 10] with limited studies in the West African sub-region [8, 9]. The few studies done in Ghana had shown low adherence to medication [16] and poor self-care behaviours [8]. The Ghanaian studies focused on only medication adherence [16] and factor’s that influence participants’ adherence to four self-care activities (diet, exercise, self-monitoring of blood glucose, and foot care) [8] without assessing adherent factors to diabetic medications. Moreover, missing in the prior studies is the link between medication adherence, self-care behaviours, and diabetes knowledge. To address this deficiency in knowledge, the present study aimed at evaluating medication adherence and self-care behaviours among patients with type 2 diabetes mellitus in Ghana.

## Methods

### Study design

This study used an analytical descriptive cross-sectional design. This design was suitable as the study was predictive in nature and examined associations [17].

### Study setting

The study was conducted at three public hospitals in the northern region of Ghana. The hospitals include one tertiary level facility and two secondary level facilities. The hospitals are situated in the Tamale Metropolis, the regional capital. These hospitals are the only facilities providing outpatient diabetes services to patients with DM in the metropolis.

### Study population

The population for the study was made up of all patients with type 2 diabetes mellitus who attended the outpatient endocrinology clinics in public hospitals within the study area. Information from the outpatient diabetes clinic registers of the various facilities estimates a total population of 1800 patients with T2DM who had been diagnosed for at least one year within the three months’ study period as the average monthly attendance was estimated at 600.

### Inclusion and exclusion criteria

The patients were qualified to be involved in the study if they; were 25 years and above and had been diagnosed with type 2 diabetes mellitus for at least one year, were on diabetes medication, and could speak, read, and write in English or Dagbani. Clients who had gestational diabetes mellitus were exempted as the condition usually remits after birth.

### Sampling and sample size determination

Consecutive sampling was used to recruit participants. The total population of patients with T2DM was estimated at 1800 for three months. This was obtained from patient records of the various hospitals. The sample size for the survey was computed according to the formula for sample size determination by Yamane [18]. The minimum required sample size was 327. A 10% non-response rate was calculated, thereby increasing it to 360. Therefore, a total of 360 patients were invited into the study for three months from August to October in 2018.

### Instrumentation

The study instrument was a structured questionnaire and consisted of four sections. The first section had demographic variables including age, gender, marital status, level of income and level of education, occupation, place of residence, and family history of diabetes. It also contained anthropometric measures such as weight, height, body mass index as well as clinical reports.

The participants’ self-care behaviours were assessed by the Summary of Diabetes Self-Care Activities (SDSCA) tool [19]. It is a self-report measure consisting of 12 items: 10 items to measure four domains of diabetes self-management, namely, diet, exercise, blood sugar testing, and foot care, and 2 items on smoking cigarettes. Participants are asked to rate how many days during the past 7 days they performed a specific self-care behaviour. The scale ranges from 0 to 7 with greater marks corresponding to better self-care. An average score is computed for each of the five domains (diet, exercise, blood glucose testing, foot care, and smoking), with greater scores indicating better diabetes self-care [19]. For this study, 10 questions from the SDSCA were applied without the question on smoking. Not smoking cigarettes was assessed as a demographic factor as previous studies assessing self-care behaviours using SDSCA did not report not smoking as a self-care behaviour [8, 20]. This tool has been previously validated and is widely used in diabetes management researches across different settings [1, 2, 7, 21].

The third section measured participants’ medication adherence using a validated medication adherence questionnaire (MAQ) developed from previous tools including; medication adherence rating scale (MARS) [22], Hill-Bone compliance to High Blood Pressure Scale [23], and Morisky medication adherence scale [24]. The MAQ was similar to the above mentioned questionnaires, but had some modifications in the vocabulary for better understanding of participants taking into consideration the local context while maintaining the relevance of each question in the original questionnaire. There were a total of 8 questions in the MAQ that assessed deliberate and non-deliberate non-adherence including reasons for non-adherence. The questions had Yes and No responses for each item. A mark of 1 was awarded for a “No” response and zero for a “Yes” response. The possible overall score for the 8-item medication adherence questionnaire ranged from zero to eight. Participants who had a total score of <6 were considered non-adherent and those whose scores were from 6–8 were categorised as adherent to medication [25]. The validity and reliability of the MAQ was ascertained before using it in this study.

Finally, the 23 item diabetes knowledge test questionnaire (DKT) developed by scholars from the University of Michigan was used to assess participants’ knowledge of diabetes [26, 27]. Every question had three answer choices with only one correct answer. Each correct response was given a score of one and an incorrect response a score of zero. The mean knowledge score was calculated and knowledge was categorized as either good or poor. A score below the mean score was coded as poor knowledge and scores above the mean were considered as good knowledge.

In line with the guidelines for translating questionnaires [28], the original English versions of the MAQ, SDSCA, and DKT instruments were forward translated into Dagbani by two bilingual translators (one had Dagbani as the native language and the other was a native English speaker). Back translation of the Dagbani version of the instruments into the English version were done by two bilingual translators who were not involved in the forward translation process; one of them was a native English speaker while the other was a native Dagbani speaker. Two of the researchers involved in the current study (RAA and AA) met with the translators and ascertained the face and content validity of instruments before being used for data collection.

### Reliability & validity

A jury panel of six individuals with expertise in nursing, endocrinology, instrument translation and validation established the face and content validity of the data collection instruments. The experts assessed the face and content validity of each item on the questionnaires by evaluating their relevance, clarity, comprehensibility, simplicity and grammatical construction. Following the content validation process, pretesting was done among 30 participants who were attending clinic in a facility with similar characteristics as the study sites before the commencement of data collection in the current study. The Cronbach alpha of the MAQ, SDSCA and DKT were 0.765, 0.69 and 0.78 respectively, indicating good internal consistency. The SDSCA has a moderate inter-item reliability (r = 0.59–0.74) [21]. Test-retest among 20 distinct participants was reported at a correlation coefficient of 0.695, demonstrating an appreciable level of stability over time (3 weeks).

### Data collection procedure

After ethical approval was obtained, four nurses were trained as research assistants to collect data. During the process of data collection, the aims and objectives and procedures of the study were explained and informed consent obtained from participants. Consent forms were signed after both formal and verbal explanations were provided; subsequently, questionnaires were handed over to participants. Between 30 and 45 minutes, participants responded to the questionnaire items and handed them to the research assistants upon completion. They were informed that they were free to withdraw from this data collection process at any time without any consequence. The research assistants were available to clarify issues participants did not understand and to provide answers to their questions in a manner that does not reveal answers to participants.

### Measures

The patients’ weight was taken with the patients wearing light clothing and without their shoes/sandals in kilograms using standardised electronic scales and height measured without shoes in centimeters. In calculating body mass index (BMI), height was converted to meters. The BMI was computed in accordance with standard guidelines by WHO and categorised as underweight, normal weight, overweight and obese [29]. Waist circumference (WC) was measured based on the WHO guidelines [30] to the nearest 1 cm using a non-stretchable fibre-glass measuring tape (Butterfly, China). Abdominal obesity was measured as a waist circumference >102 cm in men and >88 cm in women [28]. Fasting blood sugar (FBS) values for the past three months were extracted from the patients’ medical records and the mean FBS computed. The hospitals used fasting blood sugar instead of HbA1c a more accurate measure than FBS [1] due to high cost of HbA1c test in the country. Good glycaemic control was defined as FBS values less than 7.0mmol/L according to the WHO criteria. Poor glycaemic control was defined as FBS values ≥7.0mmol/L [31]. Information on complications was obtained by self-report and subsequent review of the patients medical records for confirmatory diagnosis.

### Data analysis

Of the 360 participants that were approached, 330 of them agreed to participate, resulting in a response rate of 91.7%. The 30 who refused participation expressed their disinterest in the study. There were no missing data in the collected questionnaires as the research assistants glanced through all sections to ensure their completeness. The Statistical Package for Social Sciences (SPSS) version 25.0 was used to analyse data. Numerical data were analysed using descriptive statistics such as frequencies, proportions, percentages, means and standard deviation. Categorical variables were expressed as frequencies and percentages. Measure of central tendency (means), frequencies and standard deviations were used to describe self-management practices. Chi-square analysis was performed to determine the association between categorical variables. The probability of making a type I error was set at 0.05 level for all statistical analysis. Logistic and linear regressions were used to determine factors associated with non-adherence to medication and self-care behaviours respectively.

### Ethical considerations

The study was approved by the Committee on Human Research, Publication and Ethics of the Kwame Nkrumah University of Science and Technology/Komfo Anokye Teaching Hospital (CHRPE/AP/576/18). Permission was granted by the heads of the hospitals. Participants were assured of confidentiality and anonymity of their responses.

## Results

### Socio-demographic characteristics of participants

As illustrated in Table 1, the mean (SD) age of the 330 participants was 57.5 (11.8) years; range of 25 to 91 years. Females formed more than half (n = 225, 68.2%) of the study population. Almost two-thirds, 200 (60.6%) of the participants had never had formal education and 143 (43.3%) of them were self-employed whiles 106 (32.1%) were unemployed. The majority 265 (80.3%) had family support. The majority, 223(67.6%) and 246 (74.5%) were married and Muslims respectively.

**Table 1:** Socio-demographic characteristics of participants (n = 330).

Variable	Frequency (%) Mean	Percentage	Mean (SD)
**Age**			57.5 (11.8)
**Gender**			
Male	105	31.8	
Female	225	68.2	
**Education**			
Tertiary	50	15.1	
Senior high school	33	10.0	
Junior high school	24	7.3	
Primary school	23	7.0	
No formal education	200	60.6	
**Occupation**			
Private sector employment	25	7.6	
Public sector employment	56	17.0	
Self-employment	143	43.3	
No employment	106	32.1	
**Marital status**			
Single	16	4.8	
Married	223	67.6	
Divorced	29	8.8	
Widowed	62	18.8	
**Family support**			
Yes	265	80.3	
No	65	19.7	
**Religion**			
Christian	84	25.5	
Muslim	246	74.5	
**Smoking Status**			
Smoker	10	3.0	
Non smoker	320	97.0	

### Clinical characteristics of participants

The majority of participants (74.8%) were on oral hypoglycaemic agents (OHA) (refer to Table 2). Around 39.4% had diabetes complications. The mean (SD) BMI was 26.4 (6.3) kg/m^2^. About two thirds, 68.8% of the participants were abdominally obese. About 39.4% had a family history of diabetes and 38.2% lived with diabetes for 1-3 years.

**Table 2:** Clinical characteristics of participants (n = 330).

Characteristics	Frequency	Percentage (%)
**Family history of diabetes**		
Yes	130	39.4
No	130	39.4
Don’t Know	70	21.2
**Duration of diabetes**		
1–3 years	126	38.2
4–6 years	99	30.0
7–9 years	45	13.6
10+ years	60	18.2
**Type of treatment**		
OHA	247	74.8
Insulin	33	10.0
Both OHA & Insulin	50	15.2
**Diabetic complication**		
Yes	130	39.4
No	193	58.5
Not sure	7	2.1
**Body mass index (BMI)**		
**Mean BMI (SD)**	**26.4 (6.3)**	
Obese	81	24.6
Overweight	107	32.4
Normal weight	117	35.4
Underweight	25	7.6
**Waist circumference**		
Abdominally obese	227	68.8
No abdominal obesity	103	31.2
**Glycaemic control**		
Poor	191	57.9
Good	139	42.1

### Factors associated with non-adherence to medication among participants

It was revealed that 279 (84.5%) were adherent while 51 (15.5%) were non-adherent.

Results of the logistic regression revealed that younger age and lower education were the two independent factors that were significantly associated with non-adherence to medication (see Table 3). The participants who were 70 years and above were 79% less likely to be non-adherent as compared to those below 50 years [OR = 0.21 (95%CI: 0.06–0.74), p = 0.016]. Respondents with senior high school education were 3.7 times more likely to be non-adherent than those with tertiary education [OR = 3.68 (95%CI: 1.01–13.44), p = 0.049] (Table 3).

**Table 3:** Factors associated with non-adherence to medication among participants.

Variable	Adherent n = 279 (%)	Non-adherent n = 51 (%)	Total N = 330 (%)	OR [95% CI]	p-value
**Age**					
<50 years	65 (23.3)	16 (31.4)	81 (24.6)	Ref.	
50–59 years	76 (27.2)	17 (33.3)	93 (28.2)	0.91 [0.43–1.94]	0.805
60–69 years	79 (28.3)	15 (29.4)	94 (28.5)	0.77 [0.35–1.68]	0.513
70 + years	59 (21.2)	3 (5.9)	62 (18.8)	0.21 [0.06–0.74]	**0.016**
**Gender**					
Male	90 (32.3)	15 (29.4)	105 (31.8)	Ref.	
Female	189 (67.7)	36 (70.6)	225 (68.2)	1.14 [0.60–2.19]	0.688
**Education**					
Tertiary	46 (16.4)	4 (7.8)	50 (15.1)	Ref.	
Senior High School	25 (9.0)	8 (15.7)	33 (10.0)	3.68 [1.01–13.44]	**0.049**
Junior High School	20 (7.2)	4 (7.8)	24 (7.3)	2.3 [0.52–10.12]	0.271
Primary	20 (7.2)	3 (5.9)	23 (7.0)	1.73 [0.35–8.43]	0.501
No education	168 (60.2)	32 (62.8)	200 (60.6)	2.19 [0.73–6.51]	60.158
**Marital status**					
Single	13 (4.7)	3 (5.9)	16 (4.8)	Ref	
Married	189 (67.7)	34 (66.7)	223 (67.6)	0.78 [0.21–2.88]	0.709
Divorced	23 (8.2)	6 (11.7)	29 (8.8)	1.13 [0.24–5.29]	0.876
Widowed	54 (19.4)	8 (15.7)	62 (18.8)	0.64 [0.15–2.76]	0.551
**Family support**					
Yes	224 (80.3)	41 (80.4)	265 (80.3)	Ref	
No	55 (19.7)	10 (19.6)	65 (19.7)	0.99 [0.47–2.11]	0.986
**Duration of DM**					
1-3yrs	106 (38.0)	20 (39.2)	126 (38.2)	Ref	
4-6yrs	85 (30.5)	14 (27.4)	99 (30.0)	0.87 [0.42–1.83]	0.719
7-9yrs	39 (14.0)	6 (11.8)	45 (13.6)	0.81 [0.30–2.17]	0.684
10yrs>	49 (17.5)	11 (21.6	60 (18.2)	1.19 [0.53–2.67]	0.674
**Knowledge score**					
Poor knowledge	73 (26.2)	15 (29.4)	88 (26.7)	Ref	
Good knowledge	206 (73.8)	36 (70.6)	242 (73.3)	0.85 [0.44–1.64]	0.630
**Confirmed complication**					
Yes	106 (38.0)	24 (47.1)	130 (39.4)	Ref	
No	168 (60.2)	25 (49.0)	193 (58.5)	0.65 [0.36–1.21]	0.178
Not sure	5 (1.8)	2 (3.9)	7 (2.1)	1.77 [0.32–9.66]	0.511
**Type of treatment**					
OHA	209 (74.9)	38 (74.5)	247 (74.9)	Ref	
Insulin	28 (10.0)	5 (9.8)	33 (10.0)	0.98 [0.35–2.70]	0.972
Both OHA and insulin	42 (15.1)	8 (15.7)	50 (15.1)	1.04 [0.46–2.41]	0.913
**Monthly income**					
<GHS500	203 (72.8)	32 (62.8)	235 (71.2)	Ref	
GHS 500–1000	49 (17.6)	15 (29.4)	64 (19.4)	1.94 [0.98–3.86]	0.059
GHS>1000–2000	17 (6.1)	2 (3.9)	19 (5.8)	0.75 [0.16–3.38]	0.704
GHS>2000–3000	8 (2.9)	2 (3.9)	10 (3.0)	1.59 [0.32–7.81]	0.571

NB: OHA–Oral Hypoglycaemic Agent

### Factors associated with participants’ frequency of adhering to self-management practices

Regression models for the factors associated with frequency of adherence were modelled in the form *Y* = *β*_0_ + *β*_1_*X*_1_ + *ε*_*i*_, where Y is the dependent variable, *B*_0_ is the intercept and *B*_1_ is the coefficient of the independent variable and *X*_1_ is the independent variable and *ε*_*i*_ is the error term.

In Table 4, it was revealed that number of years in school was significantly associated with patients’ frequency of adhering to diet (*Y*_*diet*_ = 3.47 − 0.14*Edu*). Female gender and non-ownership of glucometer had a decreased in SMBG frequency compared to male gender and ownership of glucometer (*Y*_*SMBG*_ = 1.97 − 0.287*FemaleSex* − 1.85*NoGlucometer*). Females had the frequency of SMBG decreased by -0.287 compared to males while those who did not own glucometer had their frequency of SMBG decreased by 1.85.

**Table 4:** Factors associated with participants’ frequency of adhering to self-management practices.

Variable	F	B	SE of beta	p-value	Partial correlation	Adjusted R square
**Diet**	6.50					**0.016**
Number of years in school		-0.141	0.055	**0.011**	-0.14	
**Exercise**	2.55					0.005
Number of years in school		-0.060	0.038	0.111	-0.09	
**SMBG**	6.33					0.016
Females (gender)		-0.287	0.114	**0.012**	-0.14	
Own glucometer		-1.855	0.047	**0.000**	-0.91	
**Foot care**	1.63					0.001
Number of years in school		-0.08	0.062	0.203	-0.07	

### Practice and knowledge score by selected variables

In Table 5, the multiple linear regression analysis using practice and knowledge as dependent variables was in the form *Y* = *β*_0_ + *β*_1_*X*_1_ + *β*_2_*X*_2_ + *β*_*n*_*X*_*n*_ + *ε*_*i*_, where Y is the dependent variable, *B*_0_ is the intercept and *B*_1_, *B*_2_, *B*_*n*_ are the coefficient of the independent variables and the the *X*_*s*_ are the independent variables and *ε*_*i*_ is the error term. The model for practice came out to be *Y*_*Practice*_ = 9.53 − 0.02*Age* + 0.18*Gender* + 1.14*EduTert* − 0.05*EduSHS* + 3.03*Knowl*. For the level of practice of self-management, tertiary education increased the level of practice by 1.14 (p = 0.041). A one-unit increase in knowledge score also increased the level of practice by 3.02 (p<0.001). The model for knowledge came out as *Y*_*Knowledge*_ = 39.0 − 0.04*Age* − 1.71*Gender* + 11.75*EduTert* + 8.06*EduSHS* + 4.10*Diet*. Tertiary education increased knowledge score by 11.75 (p<0.001) whiles SHS education increased knowledge by 8.06 (p<0.001). A unit increased in dietary practice increased knowledge by 4.10 (p<0.001). Practice and self-management are used synonymously in Table 5.

**Table 5:** Practice and knowledge score by selected variables.

	Practice score			Knowledge score		
	Unstandardized beta	Standardized beta	p-value	Unstandardized beta	Standardized beta	p-value
**Age**	-0.020	-0.067	0.191	-0.043	-0.047	0.343
**Gender**	0.181	0.023	0.658	-1.706	-0.073	0.165
**Tertiary Educ**.	1.145	0.117	**0.041**	11.75	0.387	**<0.001**
**Senior High School**	-0.050	-0.004	0.935	8.056	0.222	**<0.001**
**Knowledge score**	3.020	0.379	**<0.001**			
**Diet**				4.100	0.181	**<0.001**

## Discussion

Many researchers and health professionals have acknowledged that diabetes is a self-management disease with the onus on the patients to take care of themselves [6, 7]. Two of the most essential facets of diabetes management are medication adherence and self-care behaviours. Adherence to these aspects of care is very challenging for most patients with diabetes [7]. Previous studies have established that adherence to medication has a positive influence on glycaemic control in patients with type 2 diabetes mellitus [6, 7]. The present study assessed the prevalence of medication adherence, examined factors associated with adherence to medication and self-management behaviours among patients with type 2 diabetes mellitus.

In line with prior studies [32, 33], an overwhelming percentage of the participants were adherent to medication. This study found no statistically significant association between knowledge and medication adherence. We therefore argue that participants might have had the perception that medication is more important than other self-care behaviours for example diet and exercise to control diabetes [32, 34]. It could be assumed that clinicians probably focused more on the importance of adherence to therapeutic regimen than other self-care behaviours. Contrasting findings were reported in Cameroon [9] and Malaysia [7] whereby the majority of patients were non-adherent to medication. The study in Cameroon cited financial challenges as a reason for the low adherence with patients having to purchase expensive drugs out of pocket. Though the National Health Insurance status of participants was not assessed, its coverage on anti-diabetes medications in Ghana might have contributed to the high adherence rate of the present study population as most of the patients were not affluent.

This study observed age to be positively associated with non-adherence to medication. Younger patients were more likely to be non-adherent compared to older patients (70 years and above). Health is very valuable, and to some it may not be of utmost importance [35]. Younger adult patients may be less interested in managing their disease condition, as they may have busy schedules at work with little time to comply with healthy lifestyle and medication. Moreover, it is possible that non-adherent people simply could have died already leaving only old people that are adherent. Effective educational programs would be needed to target younger patients, as this category with early development of diabetes might have a longer life expectancy relevant to prevention of complications [35]. The current study finding is corroborating similar studies in France, and Malaysia [36, 13] where they found medication adherence to improve with increasing age. Older patients have good social support as well as better awareness of the essence of tight glycaemic control to avert complication [2]. Medication adherence also had a relationship with educational level of participants. Participants with senior high school education had 3.7 times higher odds of non-adherence compared to those with tertiary education. In this study, participants with tertiary education had higher knowledge score. The results show that patients with good practice of self-care activities was significantly associated with adherence to anti-diabetes medication. This signifies that these patients probably had a better awareness of the essence of diabetes control. Consistent with previous studies [1, 37], knowledge had a significant and positive correlation with practice of self-management. This provides a vital insight for health professionals, which signifies that they should keep educating individuals with T2DM about the consequences of and how to manage their diabetes to reduce its impact. To accomplish this, it is imperative to explore more avenues to augment the knowledge that patients already have towards their diabetes and about self-management in particular. It has been revealed from this study that patients’ number of years in school is positively correlated with regularity of adherence to dietary practice. This result concurs with data from China [38, 39] and other parts of sub Saharan Africa [40, 41]. Clients who have attained higher education are more likely to understand all aspects of recommended self-management practices including diet than their colleagues with less education [8]. This is however contrasting with findings from Greece where higher education was not associated with adherence to diet [42]. This is a concern that require attention since most of the patients in this study and earlier studies carried out in the country are often less educated [8, 37].

### Limitations of the study

The limitation of this study is that the use of self-report method to evaluate patients’ adherence to anti-diabetes medications could have resulted in overestimation or underestimation of the level of adherence. Moreover, the medication adherence questionnaire is only a surrogate for adherence, not actual medication taking behaviour. The use of consecutive sampling is an important limitation as potential participants did not have an equal chance of being included in the study.

### Implications for policy

According to a report by the Ghana Health Service, diabetes is ranked among the top 15 reasons for outpatient attendance [43]. This implies that large volumes of anti-diabetes medications are required to promote adherence to medication and for improvement in the quality of life of patients with DM. The study findings sends an important signal to policy makers to develop appropriate interventions targeting younger patients with DM to promote adherence to medication and self-care behaviours. Government needs to implement community based interventions that targets behavioural risk factors for primary prevention in the wider population.

## Conclusion

The present study findings revealed a high prevalence of 84.5% of medication adherence. The factors associated with adherence were age, education, and practice of self-care activities. Knowledge also had a significant influence on self-care practice. We recommend consistency in education to deal with issues related to self-care behaviours and medication adherence as this is necessary to enhance self-management and minimize complications. Non-adherence was associated with younger age. Interventions to improve adherence should target younger and newly diagnosed patients through aggressive counselling to address healthy self-management behaviours. Other approaches that have been used to study adherence measure validity, could be future research.

## Supporting information

S1 Data (XLSX)

S1 File (DOCX)

S2 File (DOCX)

## Abbreviations

BMI: Body mass index
DKT: Diabetes knowledge test
DM: Diabetes mellitus
JHS: Junior high school
MAQ: Medication adherence questionnaire
OHA: Oral hypoglycaemic agent
SD: Standard deviation
SDSCA: Summary of diabetes self-care activities
SHS: Senior high school
SMBG: Self-monitoring of blood glucose
T2DM: Type 2 diabetes mellitus
WC: Waist circumference
WHO: World health organization
