# Supplementary material for: Medication adherence and self-care behaviours among patients with type 2 diabetes mellitus in Ghana
Source: PLoS One. 2020 Aug 21;15(8):e0237710. doi: 10.1371/journal.pone.0237710 (PMC7446850; doi:10.1371/journal.pone.0237710)
Supplement: S1 File — (DOCX) [file pone.0237710.s002.docx]

**QUESTIONNAIRE**

**Medication Adherence and Self-care Behaviours among Patients with Type 2 Diabetes Mellitus in Ghana**

**Section I: Demographic Characteristics, Anthropometric Measures and Glycaemic control**

**Please place a tick ☑**

1. Sex Male [ ] Female [ ]
2. Age …………………
3. Education status Tertiary [ ] SHS [ ] JHS [ ] Primary [ ] No formal Education [ ]
4. Occupation Private sector employment [ ] Public sector employment [ ] Self-employed [ ] No employment [ ]
5. Marital Status single [ ] married [ ] divorced [ ] Widowed [ ]
6. Type of family Nuclear [ ] Extended [ ]
7. Do you have family support? Yes [ ] No [ ]
8. Religion Christian [ ] Muslim [ ] Traditional [ ] Other [ ] specify………
9. Ethnicity Dagomba [ ] Mamprusi [ ] gonja [ ] frafra [ ] Akan [ ] Ga [ ] Other [ ] specify………
10. Monthly income < GHS 500 [ ] GHS500-1000 [ ] GHS>1000 -2000 [ ] GHS>2000-3000 [ ] GHS>3000 [ ]
11. Residence Urban [ ] Rural [ ]
12. Family history of diabetes Yes [ ] No [ ] Don’t know [ ]
13. Do you smoke or take alcohol Yes [ ] No [ ]
14. How long have you had diabetes 1-3yrs [ ] 4-6yrs [ ] 7-9yrs [ ] 10yrs or > [ ]
15. Type of treatment [ ] Oral hypoglycaemic [ ] insulin [ ] both oral and insulin
16. Do you have medically confirmed diabetic complication? Yes [ ] No [ ] Not sure [ ]
17. If yes to question **15**, which of these confirmed complications do you have? Retinopathy [ ] Neuropathy [ ] Nephropathy [ ] Cognitive impairment [ ] Heart disease [ ] Hypertension [ ] Hypoactive Sexual arousal [ ] Diabetic foot [ ]
18. How long have you been visiting this diabetic clinic? …. ………
19. How regular do you visit the diabetic clinic? Monthly [ ] every 2months [ ] every 3months [ ] every 6months [ ] Yearly [ ] Other [ ] Specify……………

**ANTHROPOMETRIC MEASURES**

| **NO.** | **PARAMETER** | **VALUE** |
| --- | --- | --- |
| 19. | Blood Pressure (mmHg) |  |
| 20. | Waist Circumference (cm) |  |
| 21. | Weight in kilograms |  |
| 22. | Height in meters |  |
| 23. | Body mass index (BMI) |  |

**GLYCAEMIC CONTROL**

| **NO.** | **FASTING BLOOD SUGAR LEVEL** | **VALUE(mmol/dl)** |
| --- | --- | --- |
| 24. | Current month |  |
| 25. | Previous month |  |
| 26. | Previous 2months |  |
| 27. | Glycated haemoglobin (HbA1_c_) |  |

**SECTION II: PRACTICE OF SELF-MANAGEMENT ACTIVITIES**

**The questions below ask you about your diabetes self-care activities during the past 7 days. If you were sick during the past 7 days, please think back to the last 7 days that you were not sick**.

**Instruction: Please circle the right response**

**Diet**

28. How many of the last **SEVEN DAYS** have you followed a healthful eating plan?

1. 1 2 3 4 5 6 7

29. On average, over the past month, how many **DAYS PER WEEK** have you followed your eating plan?

1. 1 2 3 4 5 6 7

30. On how many of the last **SEVEN DAYS** did you eat five or more servings of fruit and vegetables?

1. 1 2 3 4 5 6 7

31. On how many of the last **SEVEN DAYS** did you eat high fat foods?

1. 1 2 3 4 5 6 7

32. On how many of the last **SEVEN DAYS** did you consume a low-sugar diet?

0 1 2 3 4 5 6 7

**Exercise**

33. On how many of the last **SEVEN DAYS** did you participate in at least 30 minutes of physical activity? (Total minutes of continuous activity, including walking)

1. 1 2 3 4 5 6 7
2. On how many of the last **SEVEN DAYS** did you participate in a specific exercise session (such as walking, biking) other than what you do around the house or as part of your work?

0 1 2 3 4 5 6 7

**Self-monitoring of blood glucose**

1. On how many of the last **SEVEN DAYS** did you test your blood sugar?
2. 1 2 3 4 5 6 7
3. On how many of the last **SEVEN DAYS** did you test your blood sugar the number of times recommended by your health care provider?

0 1 2 3 4 5 6 7

**Foot Care**

1. On how many of the last **SEVEN DAYS** did you check your feet

0 1 2 3 4 5 6 7

1. On how many of the last **SEVEN DAYS** did you inspect the inside of your shoes?
2. 1 2 3 4 5 6 7

**SECTION III: ADHERENCE TO MEDICATION USING EIGHT (8) POINT MORISKY MEDICATION ADHERENCE SCALE**

| **NO.** | **QUESTION** | **Tick☑** | |  |
| --- | --- | --- | --- | --- |
|  | **MEDICATION ADHERENCE** | **YES** | | **NO** |
| 39. | Do you ever forget to take your medicine? |  | |  |
| 40. | Do you feel careless at times about taking your medicine? |  | |  |
| 41. | Do you forget to bring along your medicine when you travel away from home? |  | |  |
| 42. | Do you stop taking your medicine because you feel sick due to side effects of the medicine? |  | |  |
| 43. | Do you decide to take less of your medicine? |  | |  |
| 44. | Do you stop taking your medicine because you feel better? |  | |  |
| 45. | Do you sometimes get annoyed that you have to keep taking your medicine every day? |  | |  |
| 46 | Do you miss taking your medicine because you run out of it at home? |  | |  |
|  |  | |  |  |

**Section IV: Diabetes Know**l**edge Test Questionnaire**

47. The diabetes diet is:

a. the way most Ghanaian people eat

b. a healthy diet for most people

c. too high in carbohydrate for most people

d. too high in protein for most people

48. Which of the following is highest in carbohydrate?

a, Baked chicken

b. Swiss cheese

c. Baked potato

d. Peanut butter

4. Which of the following is highest in fat?

a. Low fat (2%) milk

b. Orange juice

c. Corn

d. Honey

50. Which of the following is a “free food”?

a Any unsweetened food

b. Any food that has “fat free” on the label

c. Any food that has “sugar free” on the label

d. Any food that has less than 20 calories per serving

51. A1C is a measure of your average blood glucose level for the past:

a. day

b. week

c. 6-12 weeks

d. 6 months

52. Which is the best method for home glucose testing?

a. Urine testing

b. Blood testing

c. Both are equally good

53. What effect does unsweetened fruit juice have on blood glucose?

a. Lowers it

b. Raises it

c. Has no effect

54. Which should not be used to treat a low blood glucose?

a. 3 hard candies

b. 1/2 cup orange juice

c. 1 cup diet soft drink

d. 1 cup skim milk

55. For a person in good control, what effect does exercise have on blood glucose?

a. Lowers it

b. Raises it

c. Has no effect

56. What effect will an infection most likely have on blood glucose?

a. Lowers it

b. Raises it

c. Has no effect

57. The best way to take care of your feet is to:

a. look at and wash them each day

b. massage them with alcohol each day

c. soak them for one hour each day

d. buy shoes a size larger than usual

58. Eating foods lower in fat decreases your risk for:

a. nerve disease

b. kidney disease

c. heart disease

d. eye disease

5. Numbness and tingling may be symptoms of:

a. kidney disease

b. nerve disease

c. eye disease

d. liver disease

60. Which of the following is usually not associated with diabetes:

a. vision problems

b. kidney problems

c. nerve problems

d. lung problems

61. Signs of ketoacidosis (DKA) include:

a. shakiness

b. sweating

c. vomiting

d. low blood glucose

62. If you are sick with the flu, you should:

a. Take less insulin

b. Drink less liquids

c. Eat more proteins

d. Test blood glucose more often

63. If you have taken rapid-acting insulin, you are most likely to have a low blood glucose reaction in:

a. Less than 2 hours

b. 3-5 hours

c. 6-12 hours

d. More than 13 hours

64. You realize just before lunch that you forgot to take your insulin at breakfast. What should you do now?

a. Skip lunch to lower your blood glucose

b. Take the insulin that you usually take at breakfast

c. Take twice as much insulin as you usually take at breakfast

d. Check your blood glucose level to decide how much insulin to take

65. If you are beginning to have a low blood glucose reaction, you should:

a. exercise

b. lie down and rest

c. drink some juice

d. take rapid-acting insulin

66. A low blood glucose reaction may be caused by:

a. too much insulin

b. too little insulin

c. too much food

d. too little exercise

67. If you take your morning insulin but skip breakfast, your blood glucose level will usually:

a. increase

b. decrease

c. remain the same

68. High blood glucose may be caused by:

a. not enough insulin

b. skipping meals

c. delaying your snack

d. skipping your exercise

6. A low blood glucose reaction may be caused by:

a. heavy exercise

b. infection

c. overeating

d. not taking your insulin
