# Supplementary material for: Medication adherence and self-care behaviours among patients with type 2 diabetes mellitus in Ghana
Source: PLoS One. 2020 Aug 21;15(8):e0237710. doi: 10.1371/journal.pone.0237710 (PMC7446850; doi:10.1371/journal.pone.0237710)
Supplement: S2 File — (DOCX) [file pone.0237710.s003.docx]

**BOHISI**

**A Maŋ Zalisi dolibu Ni A Biԑhigu Zaŋ chaŋ barnima ban mali shigiri doro**

1. Ninsala Balibu Doo [ ] Paɤa [ ]

2. A Yuma …………………………………

3. A baŋsim Kolagi [ ] Secondary Shikuru [ ] Middle Shikuru [ ] Primary Shikuru [ ]

4. Tuma Ʒingama tuma [ ] Gomnanti tuma [ ] Amaŋmaŋ tuma [ ] N ka tuma [ ]

5. Paɤ’ lana, dakoli [ ] Paɤ’ Ʒinli [ ] ŋun yidana kani ka che o [ ]

6. Daŋ balibu Dunduŋ yini dɔɣim [ ] Dɔɣim din galinsi [ ]

7. A daŋ suŋda? Yԑlimaŋli [ ] Pa Yԑlimaŋli [ ]

8. Adiini Dolodolo [ ] Jiŋ puhira [ ]

Buɣ jԑmila [ ] Din pahi [ ] waligima ............

9. A Balli Dagomba [ ] Mamprusi [ ] Zabaɣ [ ]

Farafara [ ], Kambonsi [ ] ,Akarachi [ ], Ni bali shԑŋa

10. Goli puuni nyansim miliyon dibaanu [ ], miliyon dibaanu zaŋ chaŋ miliyon pia [ ],miliyon pia zaŋ chaŋ miliyon pishi [ ], miliyonpishi zaŋ chaŋ miliyon pishi [ ], miliyon pihita [ ]

11. A biԑhigushee? Fɔŋni [ ], Tinkpaŋ [ ]

12. A daŋ puuni Ʒi’duli mini beni Di beni [ ], Di kani [ ], M bi mi [ ]

13. A leei yurila shigaari bee a yuri dam. N yura [ ], N bi yura [ ]

14. shigiri doro ni gbaai a di yuma ala. Yuuni zaŋ chaŋ yuma ata [ ], yuma anahi ayobu [ ], yuma a poi zaŋ chaŋ awɔ [ ], yuuni pia [ ]

15. so ‘shԑŋa a ni nyԑn doli tibe? Tivaana [ ], Shiriga chibbu [ ], Pa di shԑli [ ]

16. A nyԑla ŋun dihitabili ni a mali shigiri doro? Yԑlimaŋli [ ], Pa Yԑlimaŋli [ ], N bi dihitabli [ ]

17. Ayi saɣi ti bɔhiŋ pia ni anu, dori dini ka a mali ?

Nina doro [ ], Dilidihili [ ], Tamsim [ ], Suhuni doro [ ]

18. Napuŋ shԑŋa a ni chani Ashibiti maa ni ? ……………………………………..

19. A ni chani Ashibiti maa waawaayili sham? Goli [ ], Chira ayi kam [ ], Chiraata kam [ ], chira ayobu kam [ ], yuuni kam [ ], Ni din pahi [ ], Waligima [ ].

**ZAHIMBU**

| **No.** | **Malli** | **Kalinli** |
| --- | --- | --- |
| 19. | Ʒiduli |  |
| 20. | Shaani Yalim |  |
| 21. | Di timsim ni nya sham |  |
| 22. | WaƔlim tariga |  |
| 23. | Ningbuna timsim |  |

**A Ni Gu A Shigiri Zali**

| **No.** | **Di Waɣilim** | **Kalinli** |
| --- | --- | --- |
| 24. | Tuuli goli |  |
| 25. | Chira di gari |  |
| 26. | Chira di baayi din gari |  |
| 27. | Chiraata din gari |  |

**Yaɣili : II A Maŋ Suŋsim Tuma**

Bɔhigu Din pahi Bɔhiri Ni zaŋ chaŋ A shigiri

Doro a zaɣ ni doro maa dibaapɔi din gari

Doro gbaaŋa dibaapoi din gari maa, Tahima labi nyaaŋa

dibaapɔi shԑli a daa biԑra

**ZALIGU: Boomi gili labisibu din tuhii**

28. Bakɔi puuni bula ka doli n diri bindir shԑŋa dinmali alaafee.
 Pa Shԑli (0) Ndaam (1) Ayi (2) Ata (3) Anahi (4) Anu (5) Ayobu (6) Apɔi (7)

29. Goli so ŋun gari puuni wula ka leei doli diri bindir bakɔi kam puuni

Pa Shԑli (0) Ndaam (1) Ayi (2) Ata (3) Anahi (4) Anu (5) Ayobu (6) Apɔi (7)

30. Bakɔi garila ni, Binwaka mini Ʒiԑvar shԑŋa a ni di paai balibu anu be n-gari

Pa Shԑli (0) Ndaam (1) Ayi (2) Ata (3) Anahi (4) Anu (5) Ayobu (6) Apɔi (7)

31. Bula ka a di kpam pam bakɔi din garila

Pa Shԑli (0) Ndaam (1) Ayi (2) Ata (3) Anahi (4) Anu (5) Ayobu (6) Apɔi (7)

32. Bakɔi garila puuni, Bindir’shԑŋa a ni di ka di shigiri pora?

Pa Shԑli (0) Ndaam (1) Ayi (2) Ata (3) Anahi (4) Anu (5) Ayobu (6) Apɔi (7)

33. Bula ka a choriti maŋ, ka mani minti pihita lasabu

Pa Shԑli (0) Ndaam (1) Ayi (2) Ata (3) Anahi (4) Anu (5) Ayobu (6) Apɔi (7)

34. Bakɔi din garila ni, a ni pii tuuni’shԑli amaŋa ka di paani pan tumdi tuun shԑŋa yiŋa.

Pa Shԑli (0) Ndaam (1) Ayi (2) Ata (3) Anahi (4) Anu (5) Ayobu (6) Apɔi (7)

**Zaɣ niŋbu zaŋ chaŋ a Maŋ shigiri**

35. Bakɔi din garila ni pula ka chaŋ ti zam a ʒim ?

Pa Shԑli (0) Ndaam (1) Ayi (2) Ata (3) Anahi (4) Anu (5) Ayobu (6) Apɔi (7)

36. Bakɔi din garila ni pula ka chaŋ ti zam a shigiri ka di doli alaafee baŋda ni wuhi a sham?

Pa Shԑli (0) Ndaam (1) Ayi (2) Ata (3) Anahi (4) Anu (5) Ayobu (6) Apɔi (7)

37. Bakɔi din garila ni, pula ka zamdi a waɣilim?

Pa Shԑli (0) Ndaam (1) Ayi (2) Ata (3) Anahi (4) Anu (5) Ayobu (6) Apɔi (7)

38. Bakɔi din garila ni, pula ka yuli anamda muna puuni?

Pa Shԑli (0) Ndaam (1) Ayi (2) Ata (3) Anahi (4) Anu (5) Ayobu (6) Apɔi (7)

**Yaɣili III: Timma Valibu Zalisi Dolibu**

| No. | Bɔhisi | Piima | |
| --- | --- | --- | --- |
|  | Zalisi Dolibu | Yԑlimaŋli | Pa yԑlimaŋli |
| 39. | A nami yam a tim valibu yԑla? |  |  |
| 40. | A nami niŋ zaɣ’kahi a tim valibu? |  |  |
| 41. | A nami tam a ti shԑli a vaani zaŋ go yԑla? |  |  |
| 42. | A nami che a tim valibu domi ditahi barlim na zaŋ taai? |  |  |
| 43. | A nami tԑhi ka booyi a ti vaana maa? |  |  |
| 44. | A nami che a tim valibu domi a tԑhiya ni a nya alaafee? |  |  |
| 45. | A nami niŋ suhu yiɣisili domi di kuli ʒia ni a valimi tim biԑɣu kam? |  |  |
| 46. | A nami bi vali tim maa mi domi di naai zuɣu? |  |  |

**Yaɣi: IV Shigiri baŋsim zaŋ labisi bɔhisi**

47. Bindir’shԑli din tiri shiŋiri doro n-nya

a. ninsalinima ni diri sham

b. alaafee bindirigu zaŋ ninsalinima

c. bindir’shԑŋa din tiri ninsalinima yaa ti yaɣi

d. bindir’shԑŋa din tiri ninsalinima kɔba alaafee ti yaɣi

48. Bindir dini tiri yaa gari di kpee?

a. no’shԑrili

b. waagashi

c. wuljo shԑrili

d. kpakahili

49. Bindira ŋɔ dini leei mali kpam pam?

a. bihim

b. leemukom

c. kawana

d. shiri

50. Bindira ŋɔ dini lee be a laha?

a. bindiri shԑli din ka shigiri

b. bindiri shԑli din ka kpam

c. bindiri shԑli din lama bi nyaɣisa d. bindiri shԑli din tiri alaafee

d. bindirigu din mali yaa

51. “A1C” Be mali zahidi ninsala shigiri o ni chira ata din gari.

a. dabisili

b. bakɔi

c. bakɔi pia ni ayi

d. chira ayobu

52. Wula ka leei nya niŋ baŋ shigiri zahimbu ti yiŋsi ni?

a. dulimshee

b. ʒim ni

c. di zaa viԑla

53. Doro bo ka bindir’shԑli din ka shigiri mali ti zim ni?

a. di boorili

b. di duhirili

c. di ka barna shԑli

54. Din be tu ni zaŋ tibi shigiri boobu ninsala ni?

a. leemukom kopu pirigili

b. lamalee kopu

c. kopu yin bihim din mali kpam kobga puuni vaabu yim

55. Ninvuɣ’so ŋun gu o maŋ anfaani dini ka o nyari tuma ni?

a. boobu

b. pahibu

c. di ka barna

56. Bo barna ka doro yԑn mali zaŋti shigiri?

a. boobu

b. panibu

c. di ka barna

57. So’viԑli dini tu ka nira ni tooi zam o waɣilim.

a. ka a yulli m paɣili biԑɣukam

b. ka a milli ni dam biԑɣu kam

c. ka luhili kom ni hawa yini biԑɣu kam

d. ka a da namd;a din gari a

58. Ayi diri bindirigu din ka kpam,bo n che ka di ka barina.

a. ʒi’soli doro

b. yaanina doro

c. suhuni doro

d. nina doro

59. Dilidihile nyԑla din tahiri.

a. yaanina doro

b. ʒi’soli doro

c. suhuni doro

d. nina doro

60. Doroti ŋɔ dini biԑhigu bi chaŋ ti ŋmani shigiri doro.

a. niԑlisi kalisi

b. yaanina doro

c. ʒi’soli doro

d. sapuɣi doro

61. Shigiri doro ni tahiri barna shԑŋa na ninsala ʒim puuni.

a. suhibu

b. waligu

c. tibbu

d. ʒi’duli

62. Di yi niŋ ka fiɣifiɣi gbaaga, di to ni.

a. chimi shiriga biԑla

b. yum kom biԑla

c. dim bindiri shԑŋa din ni tooi suŋ ti koba yaa

d. zammi a ʒi’duli sahakam

63. Di yi niŋ ka a chibiri shԑriga sahakam, din tooi tahi shigiri na ti a.

a. ka di be paai Hawa dibaayi

b. Hawa dibaayobu zaŋ chaŋ Hawa piani ayi

c. Hawa dibaayobu zaŋ chaŋ Hawa di baanu

d. din tooi gari Hawa pia ni ata

64. A dila wuntaŋ bindirigu naai ka teei nia be chibi a shiriga. Wula ka a yԑn niŋ?

a. ka a che bindirigu maa dibu ka di baligi a ʒi’dilli

b. ka a vali ti shԑli a ni vaani asiba

c. ka a vali ti shԑli ani vaani maa buyi

d. yulimi a sigiri maa zuɣu ka buɣisi di nit u ni vali tim maa sham

65. Di yi niŋ ka shigiri labiri nyaaŋa, di tu ni.

a. choriti a ni

b. ka a doni vuhi

c. ka a yu lamalee

d. ka chib shiri shԑli din mali yaa

66. Din baligiri shigiri yaa na n-nyԑ.

a. ka a zooi shiriga chibu

b. kashiriga chibu pora

c. ka diri pam

d. ka a maŋ choribu pora

67. Di yi niŋ ka chibi shiriga ka bi tooi di asiba bindirigu. A ʒi’duli ni zani dede.

a. di ni pahi

b. di ni labi nyaaŋa

c. di yԑn bԑla di biԑhigu

68. Di tahiri shigiri yaa na n-nyԑ.

a. ka shiriga chibu be zooi

b. ka be dira

c. ka be yuri bin nyura

d. ka be choriti a niŋ

69. Din baligiri shigiri yaa n-nyԑ.

a. ka chori ti a maŋ pampam

b. doro

c. dibu ti yaɣi

d. ka bi vaani a tim
